# Supplementary material for: Ultrafast enzymatic digestion of proteins by microdroplet mass spectrometry
Source: Nat Commun. 2020 Feb 26;11:1049. doi: 10.1038/s41467-020-14877-x (PMC7044307; doi:10.1038/s41467-020-14877-x)
Supplement: Supplementary file 1 — Supplementary Information [file 41467_2020_14877_MOESM1_ESM.pdf]

Supplementary Information

Ultrafast enzymatic digestion of proteins by microdroplet mass spectrometry

Xiaoqin Zhong,[a] *Hao Chen,*[b] *Richard N. Zare*[a,*]

[a]*Department of Chemistry, Fudan University, Shanghai, 200438, China*

[b]*Department of Chemistry and Environmental Science, New Jersey Institute of Technology, Newark, NJ 07102, USA*

**Correspondence should be addressed to Richard N. Zare*

rnz@fudan.edu.cn

Supplementary Method 1: Size distribution measurement of droplets generated by a commercial ESI sprayer and a homebuilt ESSI sprayer with a laser particle analyzer.

Laser particle analyzer: HELOS (Hi208, Sympatec GmbH, Suzhou, China).

Commercial ESI sprayer: The heated ESI source, fitted for a high-resolution mass spectrometer (LTQ Orbitrap Elite, Thermo Scientific, San Jose, CA), has a tip size of 500 µm in diameter. An aqueous solution containing 5 mM NH_4HCO_3 (pH 8) was infused with a syringe at a flow rate of 20 µL/min, with assistance of a sheath gas flow rate of 10 arbitrary units (10 psi) and a high voltage of +3 kV.

Homebuilt ESSI sprayer: from the tip of a fused silica inner capillary (148 µm o.d., 50 µm i.d., Polymicro Technologies, China), aqueous solution containing 5 mM NH_4HCO_3 (pH 8) was infused with a syringe at a flow rate of 20 µL/min, with assistance of a nebulizing gas of dry N_2 with a pressure of 120 psi and a high voltage of +3 kV.

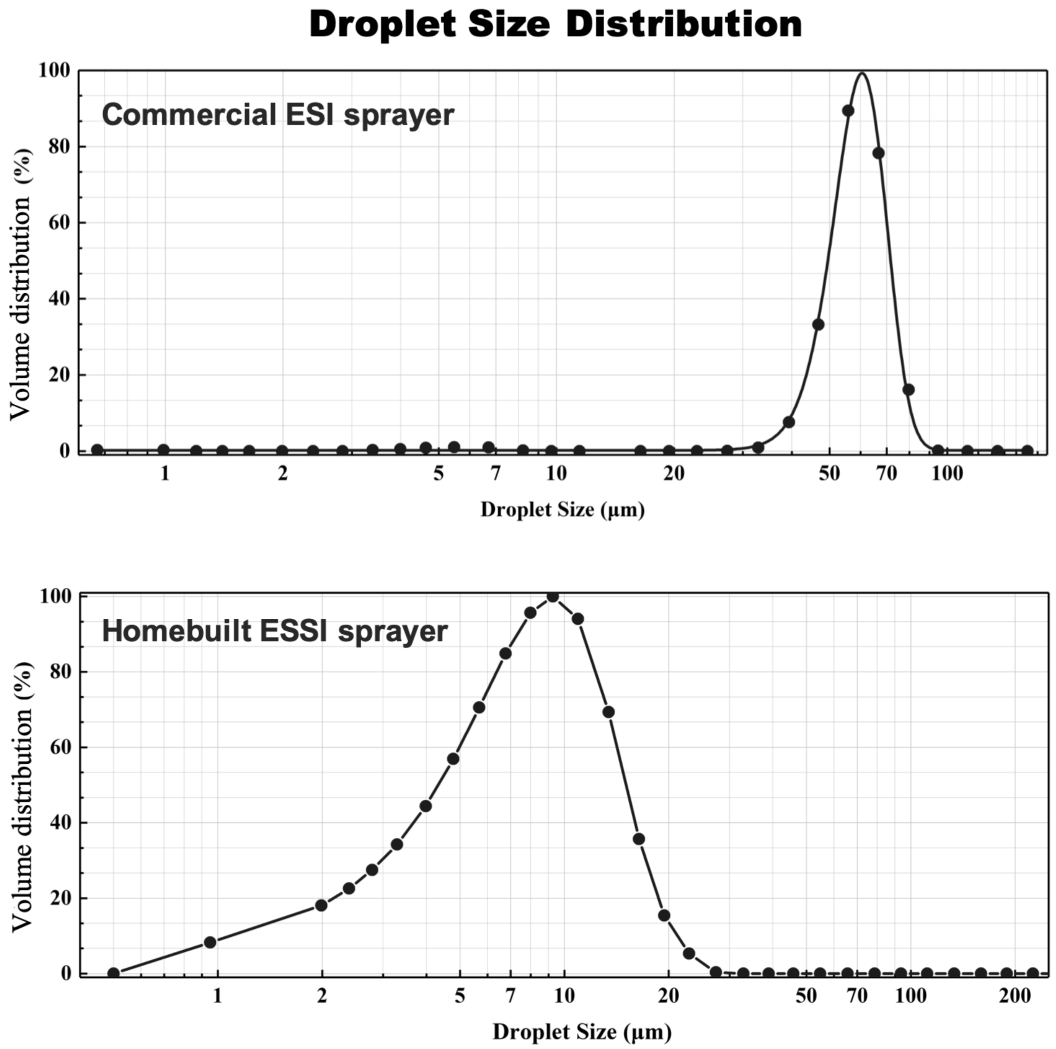

Supplementary Figure 1: Size distribution measurement of droplets generated by a commercial ESI sprayer and a homebuilt ESSI sprayer with a laser particle analyzer.

Supplementary Table 1: 26 peptide fragment peaks from the digestion of human ACTH (1-24) with ESSI-MS. A positive voltage of 3 kV was applied for the digestion and detection. The peptide sequence is SYSMEHFR|WGKPVGK|K|R|RPVK|VYP, where vertical lines indicate the expected cleavage sites for trypsin digestion.

Observed *m*/*z*		*z*	Peptide sequence
No. 1	219.1	3	(K)RRPVK(V)
2	261.8	3	(K)KRRPVK(V)
3	300.5	3	(R)WGKPVGKK(R)
4	338.8	3	(K)RRPVKVYP(-)
5	352.5	3	(-)SYSMEHFR(W)
6	380.0	5	(R)WGKPVGKKRRPVKVYP(-)
7	381.5	3	(K)KRRPVKVYP(-)
8	386.2	2	(R)WGKPVGK(K)
9	392.2	2	(K)KRRPVK(V)
10	429.7	2	(R)RPVKVYP(-)
11	450.2	2	(R)WGKPVGKK(R)
12	474.5	4	(R)WGKPVGKKRRPVKVYP(-)
13	507.8	2	(K)RRPVKVYP(-)
14	528.7	2	(R)WGKPVGKKR(R)
15	571.8	2	(K)KRRPVKVYP(-)
16	603.6	3	(-)SYSMEHFRWGKPVGK(K)
17	632.4	3	(R)WGKPVGKKRRPVKVYP(-)
18	646.3	3	(-)SYSMEHFRWGKPVGKK(R)
19	698.4	3	(-)SYSMEHFRWGKPVGKKR(R)
20	771.4	1	(R)WGKPVGK(K)
21	858.5	3	(-)SYSMEHFRWGKPVGKKRRPVK(V)
22	904.9	2	(-)SYSMEHFRWGKPVGK(K)
23	948.1	2	(R)WGKPVGKKRRPVKVYP(-)
24	968.9	2	(-)SYSMEHFRWGKPVGKK(R)
25	1047.0	2	(-)SYSMEHFRWGKPVGKKR(R)
26	1056.4	1	(-)SYSMEHFR(W)

Note: “-” means the sequence terminus.

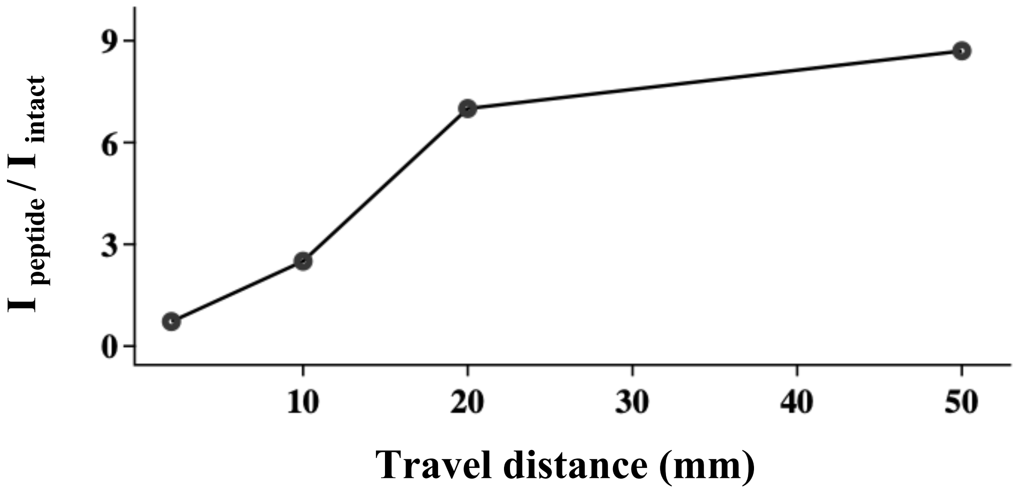

Supplementary Figure 2: Variation with microdroplet travel distance between the sprayer tip and the MS inlet of the intensity ratio of a chosen peptide at *m*/*z* 632.4, marked in pink in Fig. 2, to the sum of the intensities of all peaks from intact multicharged ACTH.

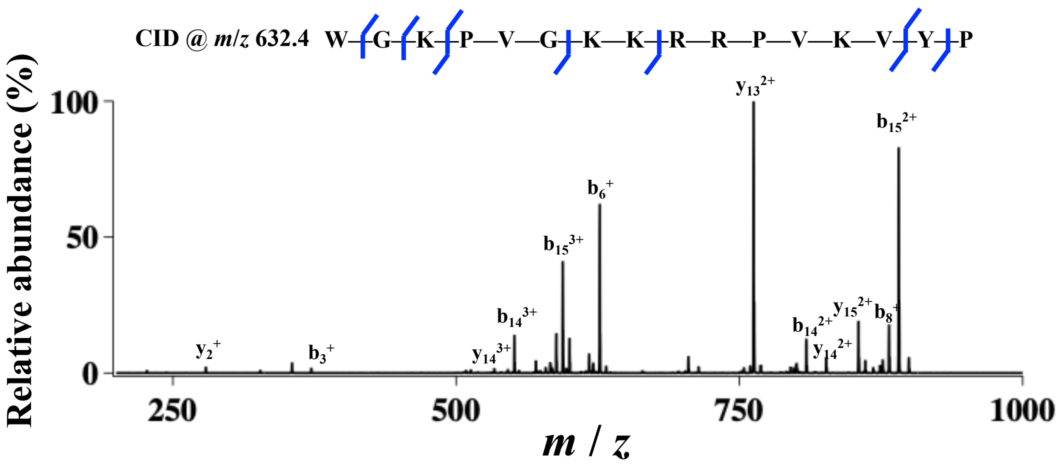

Supplementary Figure 3: Tandem MS-CID spectrum of the peptide ion at *m*/*z* 632.4 from the digest of 10-μM ACTH in 5-mM NH_4HCO_3 by ESSI-MS under a positive ion mode with a normalized energy of 25 arbitrary manufacturer's units and the mass isolation width of 1 *m*/*z*. Blue lines denote the CID fragment sites.

Supplementary Table 2: 16 peptide peaks found with the commercial ESI-MS from bulk-phase digestion of human ACTH (1-24) for 3 h at 37°C.

Observed *m*/*z*		*z*	Peptide sequence
No. 1	261.8	3	(K)KRRPVK(V)
2	300.5	3	(R)WGKPVGKK(R)
3	338.8	3	(K)RRPVKVYP(-)
4	352.5	3	(-)SYSMEHFR(W)
5	380.0	5	(R)WGKPVGKKRRPVKVYP(-)
6	381.5	3	(K)KRRPVKVYP(-)
7	386.2	2	(R)WGKPVGK(K)
8	429.7	2	(R)RPVKVYP(-)
9	450.2	2	(R)WGKPVGKK(R)
10	474.5	4	(R)WGKPVGKKRRPVKVYP(-)
11	507.8	2	(K)RRPVKVYP(-)
12	528.7	2	(R)WGKPVGKKR(R)
13	571.8	2	(K)KRRPVKVYP(-)
14	632.4	3	(R)WGKPVGKKRRPVKVYP(-)
15	646.3	3	(-)SYSMEHFRWGKPVGKK(R)
16	948.1	2	(R)WGKPVGKKRRPVKVYP(-)

Note: “-” means the sequence terminus.

Supplementary Table 3: Peptides identified from digests of myoglobin by ESSI-MS or in bulk solution followed by the commercial ESI-MS detection with a positive voltage (+3 kV).

Observed peaks, *m/z*, Th		Charge state, z	Molecular weight, Da	Peptide sequence
Microdroplet RT, < 1 ms	Bulk solution 37°C overnight			
-	368.2	2	734.4	(K)HKIPIK(Y)
423.0	-	5	2110	(K)KKGHHEAELKPLAQSHATK(H)
496.5	-	4	1982	(K)KGHHEAELKPLAQSHATK(H)
502.9	-	3	1505.7	(K)KHGTVVLTALGGILK(K)
528.5	-	4	2110	(K)KKGHHEAELKPLAQSHATK(H)
-	531.8	2	1061.6	(K)FDKFKHLK(T)
543.7	543.7	2	1085.4	(K)HLKTEAEMK(A)
551.7	-	2	1101.4	(K)HLKTEAEM^{ox}K(A)
618.6	618.6	3	1852.8	(K)GHHEAELKPLAQSHATK(H)
636.3	636.3	2	1270.6	(R)LFTGHPETLEK(F)
646.6	-	3	1936.8	(R)LFTGHPETLEKFDKFK(H)
661.7	-	3	1982.1	(K)KGHHEAELKPLAQSHATK(H)
669.0	-	3	2004	(K)FKHLKTEAEMKASEDLK(K)
674.3	-	3	2019.9	(K)FKHLKTEAEM^{Ox}KASEDLK(K)
680.9	680.9	2	1359.8	(K)ALELFRNDIAAK(Y)
-	684.3	1	683.3	(K)FDKFK(H)
689.9	689.9	2	1377.8	(K)HGTVVLTALGGILK(K)
704.3	-	3	2109.9	(K)KKGHHEAELKPLAQSHATK(H)
717.7	-	3	2150.1	(K)ASEDLKKHGTVVLTALGGILK(K)
735.5	735.5	1	734.5	(K)HKIPIK(Y)
748.4	748.4	1	747.4	(K)ALELFR(N)
752.4	-	4	3005.6	(R)LFTGHPETLEKFDKFKHLKTEAEMK(A)
753.9	753.9	2	1505.8	(K)KHGTVVLTALGGILK(K)
760.4	760.4	2	1518.8	(K)HPGDFGADAQGAM^{Ox}TK(A)
762.0	-	3	2283	(K)ALELFRNDIAAKYKELGFQG()
805.4	-	4	3217.6	(K)HLKTEAEMKASEDLKKHGTVVLTALGGILK(K)
826.4	826.4	2	1650.8	(K)ALELFRNDIAAKYK(E)
927.4	927.4	2	1852.8	(K)GHHEAELKPLAQSHATK(H)
945.2	-	4	3776.8	(R)LFTGHPETLEKFDKFKHLKTEAEMKASEDLKK(H)
969.5	-	2	1937	(R)LFTGHPETLEKFDKFK(H)
991.5	-	2	1981	(K)KGHHEAELKPLAQSHATK(H)
1002.5	-	3	3004.5	(R)LFTGHPETLEKFDKFKHLKTEAEMK(A)
1135.5	-	3	3403.5	(-)MGLSDGEWQQVLNVWGKVEADIAGHGQEVLIR(L)
-	1378.8	1	1377.8	(K)HGTVVLTALGGILK(K)

Note: “-” means the sequence terminus.

Supplementary Table 4: Peptides identified from digests of myoglobin by ESSI-MS applied with a negative voltage (-3 kV). The blue vertical lines indicate cleavage sites.

MGLSDGEWQQVLNVWGK|VEADIAGHGQEVLIR|LFTGHPETLEK|FDK|FK|HLK|TEAE
MK|ASEDLK|K|HGTVVLTALGGILK|K|K|GHHEAELKPLAQSHATK|HK|IPIK|YLEFISDAII
HVLHSK|HPGDFGADAQGAMTK|ALELFR|NDIAAK|YK|ELGFQG

Observed peaks, *m/z*, Th	Charge state, z	Molecular weight, Da	Peptide sequence
315.1	2	632.2	(R)NDIAAK(Y)
323.6	2	649.2	(K)ELGFQG(-)
352.6	2	707.2	(K)TEAEMK(A)
372.7	2	747.4	(K)ALELFR(N)
393.7	2	789.4	(K)ASEDLKK(H)
395.2	1	396.2	(K)HLK(T)
407.2	1	408.2	(K)FDK(F)
423.2	3	1272.6	(R)LFTGHPETLEK(F)
449.2	3	1350.6	(K)TEAEMKASEDLK(K)
468.3	1	469.3	(K)IPIK(Y)
504.8	3	1517.4	(K)HPGDFGADAQGAM^{Ox}TK(A)
529.8	2	1061.6	(K)FDKFKHLK(T)
541.8	2	1085.6	(K)HLKTEAEMK(A)
629.3	1	630.3	(R)NDIAAK(Y)
634.3	2	1270.6	(R)LFTGHPETLEK(F)
648.3	1	649.3	(K)ELGFQG(-)
660.3	1	661.3	(K)ASEDLK(K)
674.3	2	1350.6	(K)TEAEMKASEDLK(K)
678.8	2	1359.6	(K)ALELFRNDIAAK(Y)
682.3	1	683.3	(K)FDKFK(H)
687.9	2	1377.8	(K)HGTVVLTALGGILK(K)
733.4	1	734.4	(K)HKIPIK(Y)
746.4	1	747.4	(K)ALELFR(N)
749.8	2	1501.6	(K)HPGDFGADAQGAMTK(A)
752.3	2	1505.6	(K)KHGTVVLTALGGILK(K)
757.3	2	1516.6	(K)HPGDFGADAQGAM^{Ox}TK(A)
775.3	2	1552.6	(R)NDIAAKYKELGFQG(-)
788.4	1	789.4	(K)ASEDLKK(H)
801.9	2	1605.8	(K)VEADIAGHGQEVLIR(L)
816.4	2	1634.8	(K)HGTVVLTALGGILKKK(G)
824.4	2	1650.8	(K)ALELFRNDIAAKYK(E)
829.4	2	1660.8	(R)LFTGHPETLEKFDK(F)
849.9	4	3403.6	(-)MGLSDGEWQQVLNVWGKVEADIAGHGQEVLIR(L)
906.4	2	1814.8	(-)M$^{Met\text{-}loss}$GLSDGEWQQVLNVWGK(V)
925.4	2	1852.8	(K)GHHEAELKPLAQSHATK(H)
927.4	2	1856.8	(K)HLKTEAEMKASEDLKK(H)
939.4	1	940.4	(K)YKELGFQG(-)
967.0	2	1936	(R)LFTGHPETLEKFDKFK(H)
971.9	2	1945.8	(-)MGLSDGEWQQVLNVWGK(V)
989.5	2	1981	(K)KGHHEAELKPLAQSHATK(H)
1001.0	2	2004	(K)FKHLKTEAEMKASEDLK(K)
1060.6	1	1061.6	(K)FDKFKHLK(T)
1073.6	2	2149.2	(K)ASEDLKKHGTVVLTALGGILK(K)
1076.8	3	3233.4	(K)HLKTEAEM^{ox}KASEDLKKHGTVVLTALGGILK(K)
1084.5	1	1085.5	(K)HLKTEAEMK(A)
1121.5	3	3367.5	(K)YLEFISDAIIHVLHSKHPGDFGADAQGAMTK(A)
1133.5	3	3403.5	(-)M$^{Met\text{-}loss}$GLSDGEWQQVLNVWGKVEADIAGHGQEVLIR(L)
1140.1	2	2282.2	(K)ALELFRNDIAAKYKELGFQG(-)
1269.6	1	1270.6	(R)LFTGHPETLEK(F)
1358.7	1	1359.7	(K)ALELFRNDIAAK(Y)
1376.8	1	1377.8	(K)HGTVVLTALGGILK(K)
1500.6	1	1501.6	(K)HPGDFGADAQGAMTK(A)
1504.9	1	1505.9	(K)KHGTVVLTALGGILK(K)
1551.7	1	1552.7	(R)NDIAAKYKELGFQG(-)
1700.8	2	3403.6	(-)MGLSDGEWQQVLNVWGKVEADIAGHGQEVLIR(L)

Note: "-" means the sequence terminus.

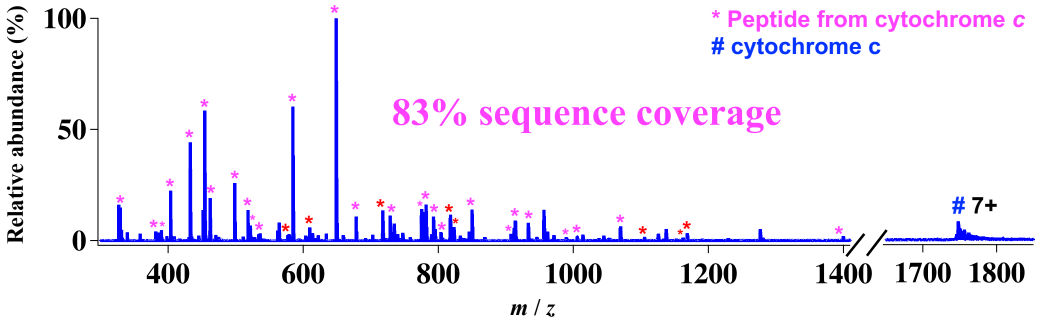

Supplementary Figure 4: Mass spectrum of peptides identified from digests of cytochrome *c* by ESSI-MS applied with a positive voltage (+3 kV). Magenta asterisks denote the peptide fragments. Blue #s mark undigested cytochrome *c* peaks.

Supplementary Table 5: Peptide list identified from digests of cytochrome *c* by ESSI-MS applied with a positive voltage (+3 kV).

Observed *m/z*		*z*	Peptide sequence
No. 1	339.7	2	(-)MGDVEK(G)
2	381.7	2	(K)KIFVQK(C)
3	390.7	2	(K)MIFAGIK(K)
4	403.7	2	(K)KYIPGTK(M)
5	432.9	3	(K)TGPNLHGLFGRK(T)
6	454.2	2	(K)MIFAGIKK(K)
7	478.9	3	(K)KGEREDLIAYLK(K)
8	486.2	3	(K)TGQAPGFSYTDANK(N)
9	518.3	2	(K)MIFAGIKKK(G)
10	521.6	3	(K)KKGEREDLIAYLK(K)
11	584.8	2	(K)TGPNLHGLFGR(K)
12	613.6	4	(K)NKGITWKEETLMEYLENPKK(Y)
13	648.8	2	(K)TGPNLHGLFGRK(T)
14	678.3	1	(K)YIPGTK(M)
15	717.9	4	(K)GITWKEETLMEYLENPKKYIPGTK(M)
16	728.8	2	(K)TGQAPGFSYTDANK(N)
17	779.4	1	(K)MIFAGIK(K)
18	781.9	2	(K)KGEREDLIAYLKK(A)
19	792.9	2	(R)KTGQAPGFSYTDANK(N)
20	803.9	2	(-)MGDVEKGKKIFVQK(C)
21	818.1	3	(K)NKGITWKEETLMEYLENPKK(Y)
22	823.4	3	(K)NKGITWKEETLM^{Ox}EYLENPKK(Y)
23	849.9	2	(K)TGQAPGFSYTDANKNK(G)
24	912.5	3	(K)TGPNLHGLFGRKTGQAPGFSYTDANK(N)
25	913.9	2	(R)KTGQAPGFSYTDANKNK(G)
26	933.5	3	(K)GITWGEETLMEYLENPKKYIPGTK(M)
27	993.2	3	(K)TGPNLHGLFGRKTGQAPGFSYTDANKNK(G)
28	1005.4	2	(K)GITWGEETLMEYLENPK(K)
29	1014.2	3	(K)NKGITWGEETLMEYLENPKKYIPGTK(M)
30	1070	2	(K)GITWGEETLMEYLENPKK(Y)
31	1162.6	2	(K)NKGITWKEETLMEYLENPK(K)
32	1168.6	1	(K)TGPNLHGLFGR(K)
33	1399.7	2	(K)GITWGEETLMEYLENPKKYIPGTK(M)

Note: "-" means the sequence terminus.

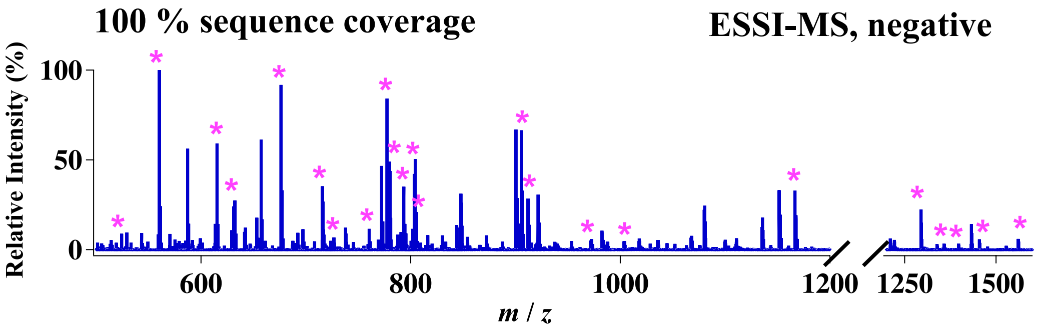

Supplementary Figure 5: Mass spectrum of peptides identified from digests of cytochrome *c* by ESSI-MS applied with a negative voltage (-3 kV). Magenta asterisks denote the peptide fragments.

Supplementary Table 6: Peptide list identified from digests of cytochrome *c* by ESSI-MS applied with a negative voltage (-3 kV).

m/z	z	Mw	sequence
524.3	1	525.3	(K)GGKHK(T)
560.27	1	561.27	(K)KATNE()
615.31	1	616.31	(K)KKGER(E)
632.38	1	633.38	(K)IFVQK(C)
676.37	1	677.37	(K)YIPGTK(M)
715.89	2	1433.78	(K)GEREDLIAYLKK(A)
726.83	2	1455.66	(K)TGQAPGFSYTDANK(N)
760.47	1	761.47	(K)KIFVQK(C)
777.44	1	778.44	(K)MIFAGIK(K)
779.94	2	1561.88	(K)KGEREDLIAYLKK(A)
790.87	2	1583.74	(R)KTGQAPGFSYTDANK(N)
801.95	2	1605.9	(-)MGDVEKGKKIFVQK(C)
804.46	1	805.46	(K)KYIPGTK(M)
847.89	2	1697.78	(K)TGQAPGFSYTDANKNK(G)
905.53	1	906.53	(K)MIFAGIKK(K)
911.94	2	1825.88	(R)KTGQAPGFSYTDANKNK(G)
972.49	2	1946.98	(K)GKKIFVQKCAQCHTVEK(G)
1003.47	2	2008.94	(K)GITWGEETLMEYLENPK(K)
1166.61	1	1167.61	(K)TGPNLHGLFGR(K)
1294.7	1	1295.7	(K)TGPNLHGLFGRK(T)
1304.69	1	1305.69	(K)GEREDLIAYLK(K)
1397.69	2	2797.38	(K)GITWGEETLMEYLENPKKYIPGTK(M)
1454.66	1	1455.66	(K)TGQAPGFSYTDANK(N)
1560.88	1	1561.88	(K)KKGEREDLIAYLK(K)
1696.8	1	1697.8	(K)TGQAPGFSYTDANKNK(G)
1944.01	1	1945.01	(K)GKKIFVQKCAQCHTVEK(G)

Note: "-" means the sequence terminus.

Supplementary Table 7: Peptide list identified from digests of cytochrome *c* and α-casein extracted from an SDS-PAGE gel by ESSI-MS applied with a positive voltage (+3 kV).

Cytochrome *c* from gel: 99% sequence coverage

Observed peaks, *m*/*z*, Th	Charge state, z	Peptide sequence
453.7	2	(K)MIFAGIKK(K)
462.2	2	(K)M^{ox}IFAGIKK(K)
482.2	2	(R)EDLIAYLK(K)
489.3	1	(K)KGER(E)
504.3	2	(-)MGDVEKGKK(I)
518.2	2	(K)MIFAGIKKK(G)
528.9	3	(R)KTGQAPGFSYTDANK(N)
536.1	4	(K)IFVQKCAQCHTVEKGGKHK(T)
562.2	1	(K)KATNE(-)
566.9	3	(K)TGQAPGFSYTDANKNK(G)
584.8	2	(K)TGPNLHGLFGR(K)
631.3	2	(K)CAQCHTVEKGGK(H)
634.3	1	(K)IFVQK(C)
678.3	1	(K)YIPGTK(M)
717.9	2	(K)GEREDLIAYLKK(A)
728.8	2	(K)TGQAPGFSYTDANK(N)
756.8	3	(K)NKGITWGEETLMEYLENPK(K)
792.8	2	(R)KTGQAPGFSYTDANK(N)
803.8	2	(-)MGDVEKGKKIFVQK(C)
812.4	2	(-)M^{ox}GDVEKGKKIFVQK(C)
849.9	2	(K)TGQAPGFSYTDANKNK(G)

Note: "-" means the sequence terminus.

αS1-casein from gel: 90.3% sequence coverage

Observed peaks, *m*/*z*, Th	Charge state, z	Peptide sequence
278.1	1	(-)MK(L)
382.7	2	(K)TTMPLW(-)
388.2	1	(R)LKK(Y)
446.5	3	(K)HIQKEDVPSER(Y)
455.7	2	(K)EGIHAQQK(E)
473.7	2	(K)EKVNELSK(D)
517.2	2	(K)NRLNFLKK(I)
547.9	3	(R)FFVAPFPEVFGKEK(V)
576.4	1	(K)HYQK(A)
587.3	3	(K)TVDMESTEVFTKKTK(L)
605.9	3	(-)MKFFIFTCLLAVALAK(N)
634.3	2	(R)YLGYLEQLLR(L)
651.6	3	(-)MKLLILTCLVAVALARPK(H)
680.4	1	(R)LKKYK(V)
692.8	2	(R)FFVAPFPEVFGK(E)
747.3	3	(R)LKKYKVPQLEIVPNSAEER(L)
748.3	1	(K)TTMPLW()
773	3	(K)EPMIGVNQELAYFYPELFR(Q)
778.3	3	(K)EPM^{ox}IGVNQELAYFYPELFR(Q)
780	3	(K)QMEAESISSSEEIVPNSVEQK(H)
784	3	(K)AMKPWIQPKTKVIPYVRYL(-)
787.2	6	(R)QFYQLDAYPSGAWYYVPLGTQYTDAPSFSDIPNPIGSENSEK(T)
803.1	4	(K)EGIHAQQKEPMIGVNQELAYFYPELFR(Q)
806.9	4	(K)EGIHAQQKEPM^{ox}IGVNQELAYFYPELFR(Q)
821.9	2	(R)FFVAPFPEVFGKEK(V)
830.9	2	(K)KTVDMESTEVFTKK(T)
880.4	2	K)TVDMESTEVFTKKTK(L)
908.4	2	(-)MKFFIFTCLLAVALAK(N)
944.2	5	(R)QFYQLDAYPSGAWYYVPLGTQYTDAPSFSDIPNPIGSENSEK(T)
976.9	2	(-)MKLLILTCLVAVALARPK(H)
1070	3	(K)EGIHAQQKEPMIGVNQELAYFYPELFR(Q)
1089.1	2	(K)VPQLEIVPNSAEERLHSMK(E)
1159	2	(K)EPMIGVNQELAYFYPELFR(Q)
1180.3	4	(R)QFYQLDAYPSGAWYYVPLGTQYTDAPSFSDIPNPIGSENSEK(T)

Note: “-” means the sequence terminus.

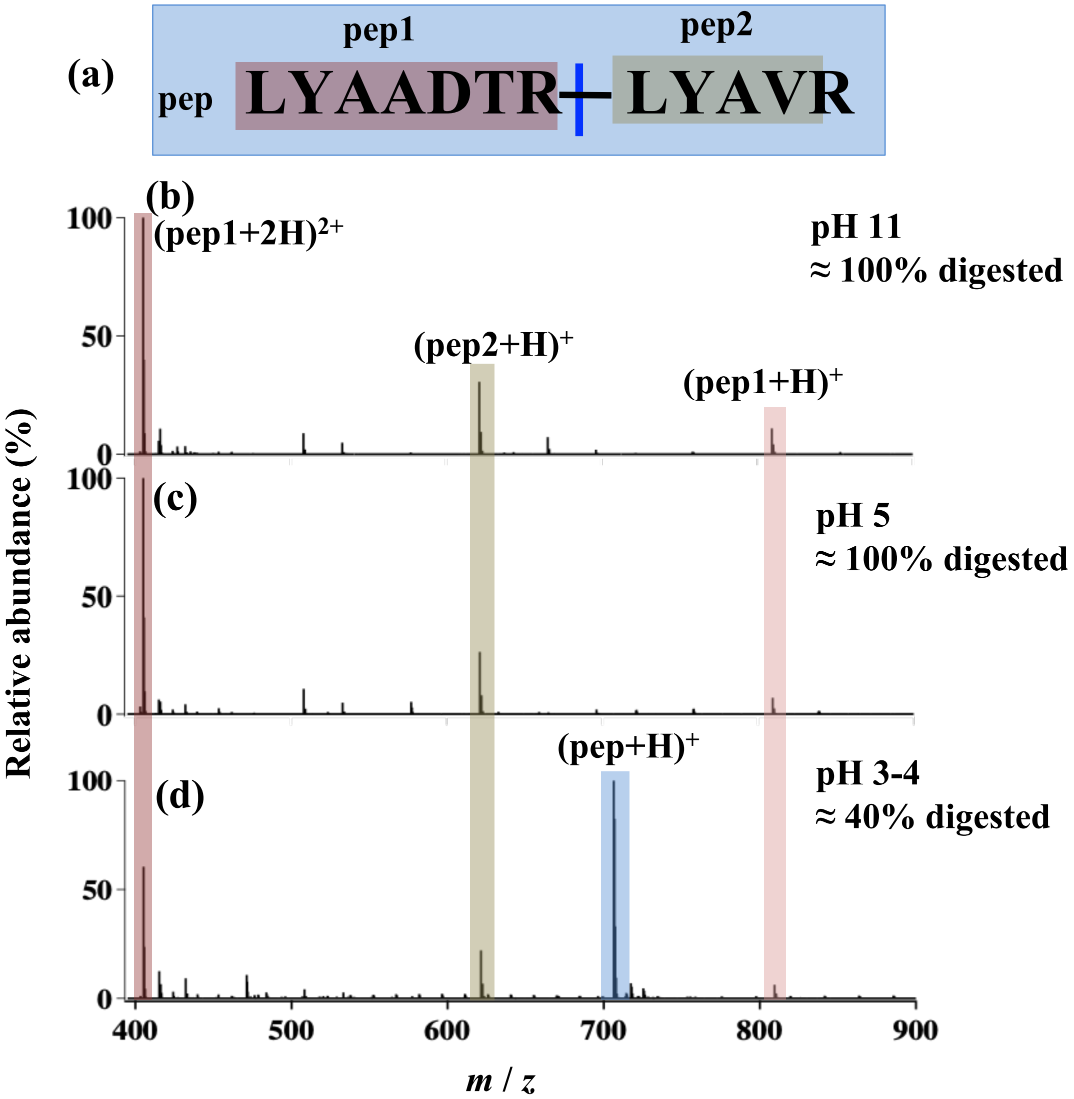

Supplementary Figure 6: Mass spectra of the digest of a synthetic peptide in 5-mM NH_4HCO_3 with different pH values by ESSI-MS: **(a)** peptide sequence, **(b)** pH 11, **(c)** pH 5, and **(d)** pH 3-4. A positive voltage (+3 kV) was applied. The blue line in the sequence marked the fragment site. The rectangles marked in various colors denote the synthetic peptide and its two fragments.

Supplementary Table 8: Peptide list identified from tryptic digests of the therapeutic antibody of trastuzumab by ESSI-MS applied with a negative voltage (-3 kV).

Light chain, 100% sequence coverage

m/z	z	sequence	modification
365.17	1	(R)GEC(-)	Carbamidomethyl
486.3	1	(K)VEIK(R)	
521.25	1	(K)SFNR(G)	
551.26	1	(R)FSGSR(S)	
558.31	1	(K)VQWK(V)	
623.27	1	(K)ADYEK(H)	
642.4	1	(K)VEIKR(T)	
690.42	1	(R)VTITCR(A)	
710.65	3	(K)VDNALQSGNSQESVTEQDSK(D)	
747.39	1	(R)VTITCR(A)	Carbamidomethyl
749.87	2	(K)DSTYSLSSTLTLSK(A)	
810.35	1	(K)SFNRGEC(-)	
867.35	1	(K)SFNRGEC(-)	Carbamidomethyl
885.47	2	(K)LLIYSASFLYSGVPSR(F)	
888.43	1	(K)ADYEKHK(V)	Carbamidomethyl
897.44	2	(K)SGTASVVCLLNNFYPR(E)	Carbamidomethyl
936.46	2	(K)VYACEVTHQGLSSPVTK(S)	Carbamidomethyl
945.94	2	(-)DIQMTQSPSSLSASVGDR(V)	Oxidation
972.01	2	(R)TVAAPSVFIFPPSDEQLK(S)	
994.5	2	(R)ASQDVNTAVAWYQQKPGK(A)	
1050.06	2	(K)RTVAAPSVFIFPPSDEQLK(S)	
1061.55	2	(K)SGTASVVCLLNNFYPREAK(V)	Carbamidomethyl
1066.98	2	(K)VDNALQSGNSQESVTEQDSK(D)	
1142.59	2	(R)ASQDVNTAVAWYQQKPGKAPK(L)	
1152.57	2	(K)LLIYSASFLYSGVPSRFSGSR(S)	
1205.57	3	(K)VDNALQSGNSQESVTEQDSKDSTYSLSSTLTLSK(A)	
1322.61	2	(K)HKVYACEVTHQGLSSPVTKSFNR(G)	Carbamidomethyl
1332.65	2	(K)SGTASVVCLLNNFYPREAKVQWK(V)	Carbamidomethyl
1337.63	2	(K)VQWKVDNALQSGNSQESVTEQDSK(D)	
1363.14	2	(K)VYACEVTHQGLSSPVTKSFNRGEC(-)	Carbamidomethyl
1386.34	3	(K)VQWKVDNALQSGNSQESVTEQDSKDSTYSLSSTLTLSK(A)	
1394.97	3	(R)SGTDFTLTISSLQPEDFATYYCQQHYTTPPTFGQGTK(V)	Carbamidomethyl
1500.75	1	(K)DSTYSLSSTLTLSK(A)	
1603.44	3	(R)SGTDFTLTISSLQPEDFATYYCQQHYTTPPTFGQGTKVEIKR(T)	Carbamidomethyl
1795.9	1	(K)SGTASVVCLLNNFYPR(E)	Carbamidomethyl
1809.36	2	(K)VDNALQSGNSQESVTEQDSKDSTYSLSSTLTLSK(A)	
1860.96	2	(R)TVAAPSVFIFPPSDEQLKSGTASVVCLLNNFYPR(E)	Carbamidomethyl
1873.93	1	(K)VYACEVTHQGLSSPVTK(S)	Carbamidomethyl
1944.03	1	(R)TVAAPSVFIFPPSDEQLK(S)	
2093.47	2	(R)SGTDFTLTISSLQPEDFATYYCQQHYTTPPTFGQGTK(V)	Carbamidomethyl

Note: “-” means the sequence terminus.

Heavy chain, 86% sequence coverage

m/z	z	sequence	modification
359.2	1	(K)VDK(K)	
437.21	1	(K)EYK(C)	
445.24	1	(K)VSNK(A)	
446.27	1	(K)TISK(A)	
455.24	1	(K)GQPR(E)	
470.27	1	(K)VEPK(S)	
487.29	1	(K)VDKK(V)	
498.27	1	(R)QAPGK(G)	
499.3	1	(K)TKPR(E)	
573.33	1	(K)LTVDK(S)	
598.36	1	(K)KVEPK(S)	
623.3	3	(K)TTPPVLDSDGSFFLYSK(L)	
626	3	(-)EVQLVESGGGLVQPGGSLR(L)	
634.78	4	(K)GFYPSDIAVEWESNGQPENNYK(T)	
635.3	1	(R)EEMTK(N)	
640.33	3	(K)AKGQPREPQVYTLPPSR(E)	
641.83	2	(R)EPQVYTLPPSR(E)	
645.39	1	(K)TISKAK(G)	
654.37	2	(R)QAPGKGLEWVAR(I)	
662.32	2	(K)NTAYLQMNSLR(A)	Oxidation
665.78	2	(R)AEDTAVYYCSR(W)	Carbamidomethyl
680.33	1	(R)YADSVK(G)	
712.01	3	(R)TPEVTCVVVDVSHEDPEVK(F)	Carbamidomethyl
786.42	1	(K)SLSLSPGK(-)	
816.46	1	(K)LTVDKSR(W)	
828.44	1	(K)GLEWVAR(I)	
836.49	1	(K)ALPAPIEK(T)	
848.39	2	(K)VSNKALPAPIEKTISK(A)	
847.04	3	(K)GFYPSDIAVEWESNGQPENNYK(T)	
850.42	1	(K)DTLMISR(T)	
868.35	2	(R)EEMTKNQVSLTCLVK(G)	Oxidation
898.43	2	(R)EEMTKNQVSLTCLVK(G)	Carbamidomethyl & Oxidation
902.5	2	(R)VVSVLTVLHQDWLNGK(E)	
935.46	2	(K)TTPPVLDSDGSFFLYSK(L)	
940	2	(-)EVQLVESGGGLVQPGGSLR(L)	
961	2	(K)AKGQPREPQVYTLPPSR(E)	
967	1	(R)FTISADTSK(N)	
979.98	2	(R)IYPTNGYTRYADSVKGR(F)	
990.47	2	(K)CKVSNKALPAPIEKTISK(A)	Carbamidomethyl
1031.51	1	(K)KVEPKSCDK(T)	
1068.51	2	(R)TPEVTCVVVDVSHEDPEVK(F)	Carbamidomethyl
1082.53	1	(R)IYPTNGYTR(Y)	
1087.54	1	(K)DTYIHWVR(Q)	
1089.54	2	(R)LSCAASGFNIKDTYIHWVR(Q)	
1091	2	(K)CKVSNKALPAPIEKTISKAK(G)	Carbamidomethyl
1088.54	1	(K)KVEPKSCDK(T)	Carbamidomethyl
1113.1	2	(R)VVSVLTVLHQDWLNGKEYK(C)	
1159.62	1	(K)NQVSLTCLVK(G)	Carbamidomethyl
1165.58	1	(R)LSCAASGFNIK(D)	Carbamidomethyl
1180.6	1	(K)GRFTISADTSK(N)	
1184.64	1	(K)GPSVFPLAPSSK(S)	
1200.63	2	(K)EYKCKVSNKALPAPIEKTISK(A)	Carbamidomethyl
1271.06	2	(K)GFYPSDIAVEWESNGQPENNYK(T)	
1279.06	2	(K)GLEWVARIYPTNGYTRYADSVK(G)	
1284.67	1	(R)EPQVYTLPPSR(E)	
1309.71	1	(R)QAPGKGLEWVAR(I)	
1319.67	1	(K)STSGGTAALGCLVK(D)	Carbamidomethyl
1325.64	1	(K)NTAYLQMNSLR(A)	Oxidation
1332.56	1	(R)AEDTAVYYCSR(W)	Carbamidomethyl
1391.13	2	(R)WGGDGFYAMDYWGQGTLVTVSSASTK(G)	
1399.68	2	(R)WQQGNVFSCSVMHEALHNHYTQK(S)	Carbamidomethyl
1420.64	2	(K)THTCPPCPAPELLGGPSVFLFPPKPK(D)	
1453.69	2	(R)IYPTNGYTRYADSVKGRFTISADTSK(N)	
1566.74	2	(R)YADSVKGRFTISADTSKNTAYLQMNSLR(A)	
1676.8	1	(K)FNWYVDGVEVHNAK(T)	
1697.78	2	(R)EEQYNSTYRVVSVLTVLHQDWLNGKEYK(C)	
1763.37	2	(R)WQQGNVFSCSVMHEALHNHYTQKSLSLSPGK(-)	Oxidation
1806	1	(R)VVSVLTVLHQDWLNGK(E)	
1872.92	1	(K)TTPPVLDSDGSFFLYSK(L)	
1880.01	1	(-)EVQLVESGGGLVQPGGSLR(L)	
1959.97	1	(R)IYPTNGYTRYADSVKGR(F)	

Note: “-” means the sequence terminus.

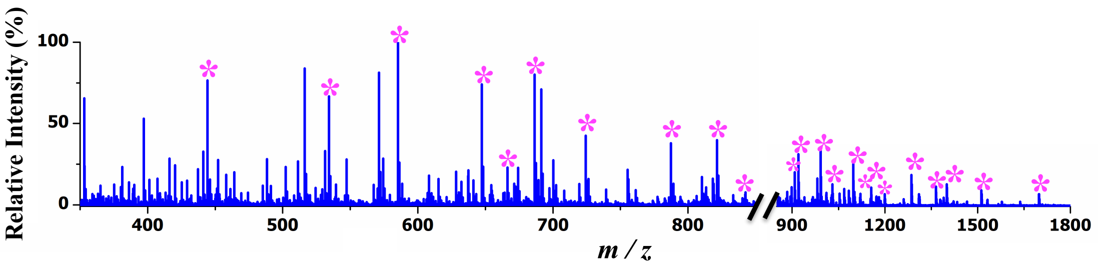

Supplementary Figure 7: Peptides identified from elastase digests of the heavy chain of therapeutic antibody of trastuzumab by ESSI-MS applied with a negative voltage (-3 kV). Magenta asterisks denote the peptide fragments.

Supplementary Table 9: Peptides identified from elastase digests of the heavy chain of therapeutic antibody of trastuzumab by ESSI-MS applied with a negative voltage (-3 kV).

m/z	*z*	Mw	Sequence
444.25	1	445.25	(A)VLQS(S)
534.31	1	535.31	(V)SLTCL(V)
585.33	1	586.33	(-)EVQLV(E)
647.36	1	648.36	(A)RIYPT(N)
686.38	1	687.38	(S)SVVTVPS(S)
724.36	1	725.36	(S)SGLYSLS(S)
654.35	2	1310.7	(V)RQAPGKGLEWVA(R)
787.4	1	788.4	(I)SRTPEVT(C)
821.5	1	822.5	(S)NKALPAPI(E)
842.38	1	843.38	(T)NGYTRYA(D)
908.53	1	909.53	(V)SNKALPAPI(E)
920.42	1	921.42	(V)SWNSGALTS(G)
992.48	1	993.48	(V)KDYFPEPV(T)
1030.57	1	1031.57	(A)STKGPSVFPL(A)
1097.61	1	1098.61	(T)KGPSVFPLAPS(S)
1119.54	1	1120.54	(S)RWQQGNVFS(C)
1155.58	1	1156.58	(V)ESGGGLVQPGGSL(R)
1309.71	1	1310.71	(V)RQAPGKGLEWVA(R)
1364.67	1	1365.67	(L)GCLVKDYFPEPV(T)
1399.81	1	1400.81	(S)VFLFPPKPKDTL(M)
1577.79	1	1578.79	(A)SGFNIKDTYIHWV(R)
1697.94	1	1698.94	(L)LGGPSVFLFPPKPKDT(L)

Note: “-” means the sequence terminus.

Heavy chain: 74% sequence coverage

EVQLVESGGGLVQPGGSLRLSCAASGFNIKDTYIHWVRQAPGKGLEWVARIYPTNGYTRYADSV
KGRFTISADTSKNTAYLQMNSLRAEDTAVYYCSRWGGDGFYAMDYWGQGTLVTVSSASTKGPS
VFPLAPSSKSTSGGTAALGCLVKDYFPEPVTVSWNSGALTSGVHTFPAVLQSSGLYSLSSVVTVPS
SSLGTQTYICNVNHKPSNTKVDKKVEPKSCDKTHTCPPCPAPELLGGPSVFLFPPKPKDTLMISRT
PEVTCVVVDVSHEDPEVKFNWYVDGVEVHNAKTKPREEQYNSTYRVVSVLTVLHQDWLNGKE
YKCKVSNKALPAPIEKTISKAKGQPREPQVYTLPPSREEMTKNQVSLTCLVKGFYPSDIAVEWES
NGQPENNYKTTPPVLDSDGSFFLYSKLTVDKSRWQQGNVFSCSVMHEALHNHYTQKSLSLSPGK

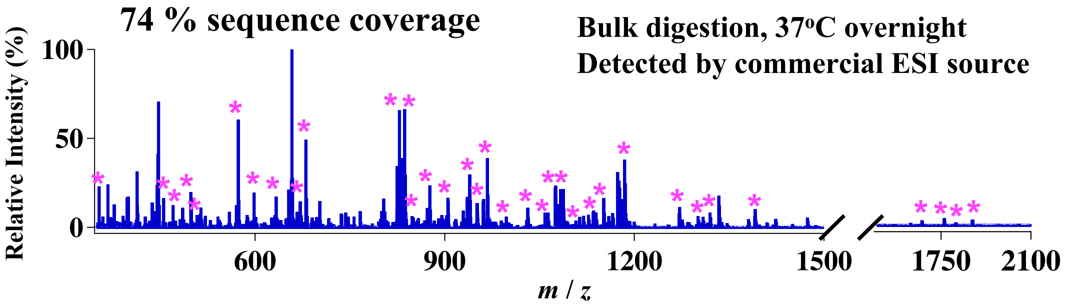

Supplementary Figure 8: Peptides identified with the commercial ESI-MS under a negative voltage (-3 kV) from tryptic digests of the heavy chain of therapeutic antibody of trastuzumab in bulk overnight at 37°C. Magenta asterisks denote the peptide fragments. The sequence marked in magenta was found from the tryptic digests in bulk and the left ones in black were lost.

Supplementary Table 10: Peptides identified with the commercial ESI-MS under a negative voltage (-3 kV) from tryptic digests of the heavy chain of therapeutic antibody of trastuzumab in bulk overnight at 37°C.

m/z	z	Mw	sequence	modification
359.2	1	360.2	(K)VDK(K)	
455.24	1	456.24	(K)GQPR(E)	
470.27	1	471.27	(K)VEPK(S)	
498.27	1	499.27	(R)QAPGK(G)	
499.3	1	500.3	(K)TKPR(E)	
507.19	1	508.19	(K)SCDK(T)	Carbamidomethyl
573.33	1	574.33	(K)LTVDK(S)	
598.36	1	599.36	(K)KVEPK(S)	
626	3	1881	(-)EVQLVESGGGLVQPGGSLR(L)	
665.78	2	1333.56	(R)AEDTAVYYCSR(W)	Carbamidomethyl
680.33	1	681.33	(R)YADSVK(G)	
828.44	1	829.44	(K)GLEWVAR(I)	
833.42	1	834.42	(K)DTLMISR(T)	
836.49	1	837.49	(K)ALPAPIEK(T)	
848.39	2	1698.78	(K)VSNKALPAPIEKTISK(A)	
850.42	1	851.42	(K)DTLMISR(T)	
868.35	2	1738.7	(R)EEMTKNQVSLTCLVK(G)	Oxidation
896.4	2	1794.8	(R)EEMTKNQVSLTCLVK(G)	Carbamidomethyl & Oxidation
902.5	2	1807	(R)VVSVLTVLHQDWLNGK(E)	
935.46	2	1872.92	(K)TTPPVLDSDGSFFLYSK(L)	
940	2	1882	(-)EVQLVESGGGLVQPGGSLR(L)	
950.96	2	1903.92	(R)EPQVYTLPPSREEMTK(N)	
961	2	1924	(K)AKGQPREPQVYTLPPSR(E)	
967	1	968	(R)FTISADTSK(N)	
979.98	2	1961.96	(R)IYPTNGYTRYADSVKGR(F)	
990.47	2	1982.94	(K)CKVSNKALPAPIEKTISK(A)	Carbamidomethyl
1031.51	1	1032.51	(K)KVEPKSCDK(T)	
1083.51	1	1084.51	(R)IYPTNGYTR(Y)	
1087.54	1	1088.54	(K)DTYIHWVR(Q)	
1107.48	1	1108.48	(R)LSCAASGFNIK(D)	
1113.1	2	2228.2	(R)VVSVLTVLHQDWLNGKEYK(C)	
1159.62	1	1160.62	(K)NQVSLTCLVK(G)	Carbamidomethyl
1165.58	1	1166.58	(R)LSCAASGFNIK(D)	Carbamidomethyl
1184.64	1	1185.64	(K)GPSVFPLAPSSK(S)	
1271.06	2	2544.12	(K)GFYPSDIAVEWESNGQPENNYK(T)	
1284.67	1	1285.67	(R)EPQVYTLPPSR(E)	
1308.64	1	1309.64	(K)NTAYLQMNSLR(A)	
1319.67	1	1320.67	(K)STSGGTAALGCLVK(D)	Carbamidomethyl
1325.64	1	1326.64	(K)NTAYLQMNSLR(A)	Oxidation
1391.13	2	2784.26	(R)WGGDGFYAMDYWGQGTLVTVSSASTK(G)	
1675.79	1	1676.79	(K)FNWYVDGVEVHNAK(T)	
1763.37	2	3528.74	(R)WQQGNVFSCSVMHEALHNHYTQKSLSLSPGK(-)	Oxidation
1806	1	1807	(R)VVSVLTVLHQDWLNGK(E)	
1872.92	1	1873.92	(K)TTPPVLDSDGSFFLYSK(L)	

Note: "-" means the sequence terminus.

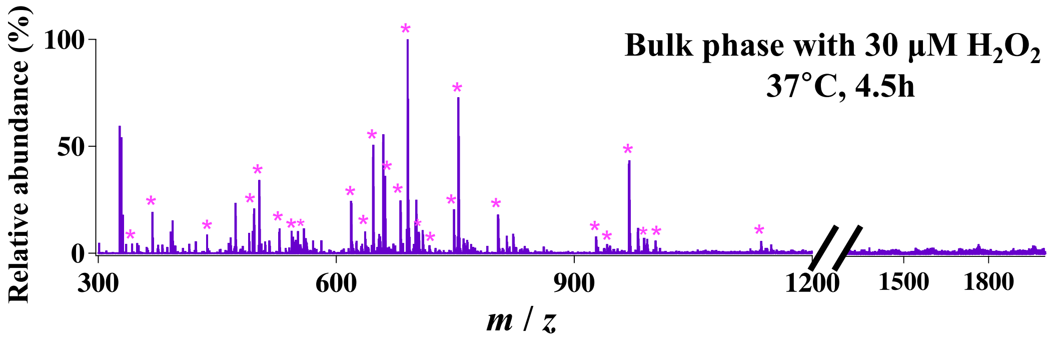

Supplementary Figure 9: Commercial ESI-MS (+3 kV) analysis of myoglobin digests in bulk phase (5 mM NH_4HCO_3, pH 8) containing 30 µM H_2O_2, at 37°C for 4.5 h. Magenta asterisks denote the peptide fragments.

Supplementary Table 11: Commercial ESI-MS (+3 kV) analysis of myoglobin digests in bulk phase (5 mM NH_4HCO_3, pH 8) containing 30 µM H_2O_2, at 37°C for 4.5h.

**Observed peaks, *m*/*z*, Th**	**Charge state, z**	**Peptide sequence**
342.7	2	(K)FDKFK(H)
368.2	2	(K)HKIPIK(Y)
423	5	(K)KKGHHEAELKPLAQSHATK(H)
496.5	4	(K)KGHHEAELKPLAQSHATK(H)
502.9	3	(K)KHGTVVLTALGGILK(K)
528.5	4	(K)KKGHHEAELKPLAQSHATK(H)
543.8	2	(K)HLKTEAEMK(A)
551.7	2	(K)HLKTEAEM^{ox}K(A)
618.6	3	(K)GHHEAELKPLAQSHATK(H)
636.3	2	(R)LFTGHPETLEK(F)
646.6	3	(R)LFTGHPETLEKFDKFK(H)
661.7	3	(K)KGHHEAELKPLAQSHATK(H)
680.8	2	(K)ALELFRNDIAAK(Y)
689.9	2	(K)HGTVVLTALGGILK(K)
704	3	(K)KKGHHEAELKPLAQSHATK(H)
717.4	3	(K)ASEDLKKHGTVVLTALGGILK(K)
748.4	1	(K)ALELFR(N)
753.9	2	(K)KHGTVVLTALGGILK(K)
803.9	2	(K)VEADIAGHGQEVLIR(L)
927.5	2	(K)GHHEAELKPLAQSHATK(H)
945	4	(R)LFTGHPETLEKFDKFKHLKTEAEMKASEDLKK(H)
969.5	2	(R)LFTGHPETLEKFDKFK(H)
992	2	(K)KGHHEAELKPLAQSHATK(H)
1002.5	3	(R)LFTGHPETLEKFDKFKHLKTEAEMK(A)
1135.5	3	(-)MGLSDGEWQQVLNVWGKVEADIAGHGQEVLIR(L)

Note: "-" means the sequence terminus.

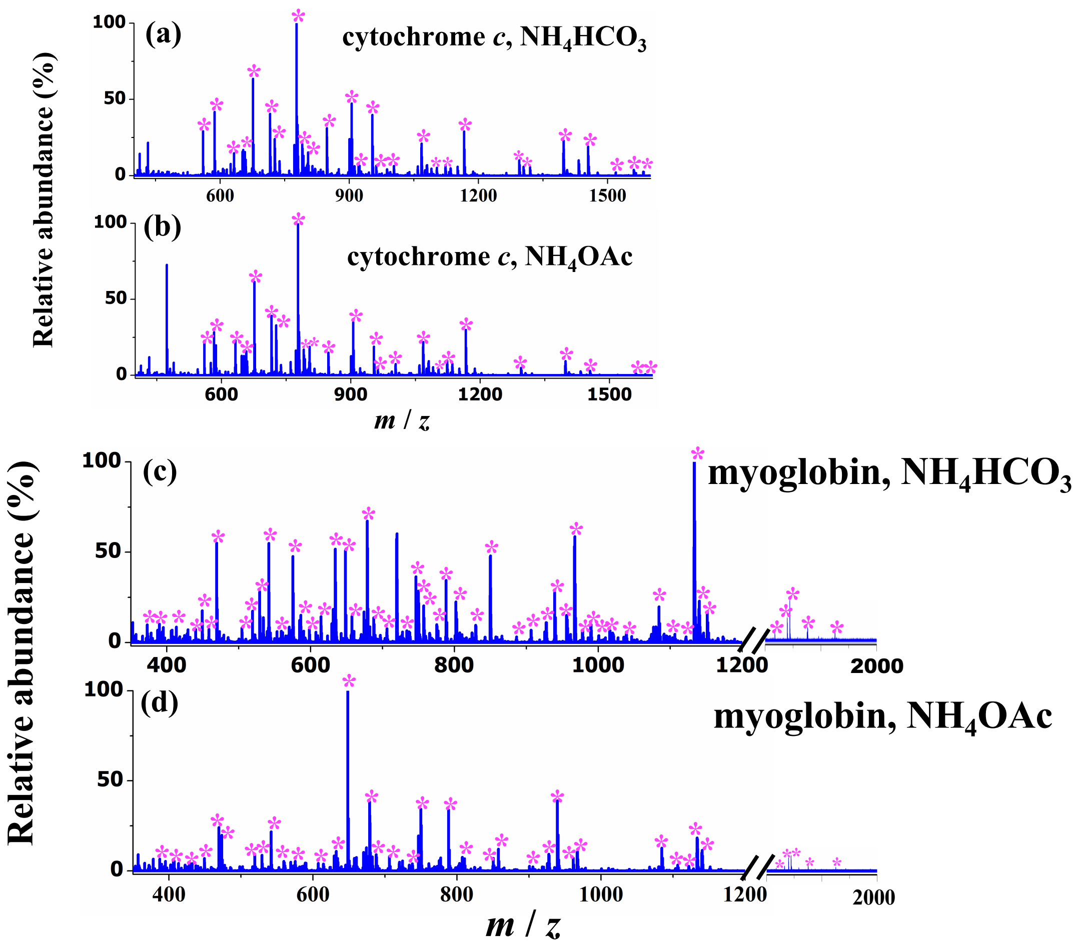

Supplementary Figure 10: ESSI-MS (-3 kV) analysis of protein digests in different buffer solution: **(a)** 10 µM cytochrome *c* in 5 mM ammonium bicarbonate (NH_4HCO_3, pH 8), **(b)** 10 µM cytochrome *c* in 5 mM ammonium acetate (NH_4OAc, pH 8), **(c)** 10 µM myoglobin in 5 mM NH_4HCO_3 (pH 8), and **(d)** 10 µM myoglobin in 5 mM NH_4OAc (pH 8). Trypsin concentration was always kept as 5 µg/mL in respective buffer solution. Magenta asterisks denote the peptide fragments.
