# Supplementary material for: Ultrafast enzymatic digestion of proteins by microdroplet mass spectrometry
Source: Nat Commun. 2020 Feb 26;11:1049. doi: 10.1038/s41467-020-14877-x (PMC7044307; doi:10.1038/s41467-020-14877-x)
Supplement: Supplementary file 3 — Reporting Summary [file 41467_2020_14877_MOESM3_ESM.pdf]

nature research

Corresponding author(s): Richard N. Zare

Last updated by author(s): Jan 11, 2020

Reporting Summary

Nature Research wishes to improve the reproducibility of the work that we publish. This form provides structure for consistency and transparency in reporting. For further information on Nature Research policies, see Authors & Referees and the Editorial Policy Checklist.

Please do not complete any field with "not applicable" or n/a. Refer to the help text for what text to use if an item is not relevant to your study.
For final submission: please carefully check your responses for accuracy; you will not be able to make changes later.

Statistics

For all statistical analyses, confirm that the following items are present in the figure legend, table legend, main text, or Methods section.

n/a	Confirmed	
☒	☐	The exact sample size (*n*) for each experimental group/condition, given as a discrete number and unit of measurement
☒	☐	A statement on whether measurements were taken from distinct samples or whether the same sample was measured repeatedly
☒	☐	The statistical test(s) used AND whether they are one- or two-sided<br>*Only common tests should be described solely by name; describe more complex techniques in the Methods section.*
☒	☐	A description of all covariates tested
☒	☐	A description of any assumptions or corrections, such as tests of normality and adjustment for multiple comparisons
☒	☐	A full description of the statistical parameters including central tendency (e.g. means) or other basic estimates (e.g. regression coefficient) AND variation (e.g. standard deviation) or associated estimates of uncertainty (e.g. confidence intervals)
☒	☐	For null hypothesis testing, the test statistic (e.g. *F*, *t*, *r*) with confidence intervals, effect sizes, degrees of freedom and *P* value noted<br>*Give P values as exact values whenever suitable.*
☒	☐	For Bayesian analysis, information on the choice of priors and Markov chain Monte Carlo settings
☒	☐	For hierarchical and complex designs, identification of the appropriate level for tests and full reporting of outcomes
☒	☐	Estimates of effect sizes (e.g. Cohen's *d*, Pearson's *r*), indicating how they were calculated

Our web collection on statistics for biologists contains articles on many of the points above.

Software and code

Policy information about availability of computer code

Data collection	Data collection was by the commercial Xcalibur™ Software from Thermo Fisher Scientific.
Data analysis	Data analysis and conversion into exact mass list were performed by Xcalibur Qual Browser (ThermoFisher Scientific, San Jose, CA). The mass spectra were plotted by IGOR Pro (Version 6.00 for Macintosh, WaveMetrics, Lake Oswego, OR, USA).MS-digest program from Protein Prospector version 5.19.1 (University of California, San Francisco, CA, USA) was applied for an in silico digest of the protein of interest. Protein accession code and database are obtained from UniprotKB. The search parameters with MS-digest program are set as following: trypsin digestion, three maximum missed cleavages, variable modification of oxidation for cytochrome c, myoglobin and α-casein, variable modifications of oxidation and carbamidomethyl (C) for trastuzumab antibody. 5 ppm is considered as the mass tolerance between the experimental and theoretical mass.

For manuscripts utilizing custom algorithms or software that are central to the research but not yet described in published literature, software must be made available to editors/reviewers. We strongly encourage code deposition in a community repository (e.g. GitHub). See the Nature Research guidelines for submitting code & software for further information.

Data

Policy information about availability of data

All manuscripts must include a data availability statement. This statement should provide the following information, where applicable:

- Accession codes, unique identifiers, or web links for publicly available datasets
- A list of figures that have associated raw data
- A description of any restrictions on data availability

Data Availability
There are no restrictions for the raw data associated with the figures presented and they are available from the corresponding authors on reasonable request. We have given accession codes for the proteins we have studied in the main text.

Field-specific reporting

Please select the one below that is the best fit for your research. If you are not sure, read the appropriate sections before making your selection.

- [x] Life sciences
- [ ] Behavioural & social sciences
- [ ] Ecological, evolutionary & environmental sciences

Life sciences study design

All studies must disclose on these points even when the disclosure is negative.

Sample size	The manuscript is focused on acceleration of protein digestion for sequencing. 3 peptides, 4 proteins and one therapeutic antibody were used as the models to test the acceleration effect of of microdroplet MS on proteolytic digestion.
Data exclusions	There is no data exclusion.
Replication	Each sample was tested more than 3 technical replicates to verify the reproducibility of the acceleration effect of microdroplet MS on proteolytic digestion.
Randomization	Not applicable. We compare the results obtained by our method with those obtained by a conventional method, which incubates bulk solution containing protein and trypsin overnight at 37 degree. The results from in silico digestion by MS-digest program from Protein Prospector version 5.19.1 (University of California, San Francisco, CA, USA) are applied to verify the peptides obtained from microdroplet digests or bulk digests.
Blinding	Not applicable. We describe a new method for protein digestion. The results were verified by comparison to the ones obtained with in silico digestion by MS-digest program from Protein Prospector version 5.19.1 (University of California, San Francisco, CA, USA).

Reporting for specific materials, systems and methods

We require information from authors about some types of materials, experimental systems and methods used in many studies. Here, indicate whether each material, system or method listed is relevant to your study. If you are not sure if a list item applies to your research, read the appropriate section before selecting a response.

Materials & experimental systems

n/a	Involved in the study	
☐	☒	Antibodies
☒	☐	Eukaryotic cell lines
☒	☐	Palaeontology
☒	☐	Animals and other organisms
☒	☐	Human research participants
☒	☐	Clinical data

Methods

n/a	Involved in the study	
☒	☐	ChIP-seq
☒	☐	Flow cytometry
☒	☐	MRI-based neuroimaging

Antibodies

Antibodies used	Trastuzumab (accession number: P04626, MedChemExpress, USA) is a humanized monoclonal antibody that has been clinically used to treat patients with invasive breast cancers and sold under a brand name Herceptin among others.
Validation	Because trastuzumab is commercially obtained from the company of MedChemExpress in USA. This work is focused on digesting the antibody with microdroplet-MS to confirm its sequence and the results matched well with its theoretical sequence provided commercially. So we didn't perform any validation for its species and biological application.

This checklist template is licensed under a Creative Commons Attribution 4.0 International License, which permits use, sharing, adaptation, distribution and reproduction in any medium or format, as long as you give appropriate credit to the original author(s) and the source, provide a link to the Creative Commons license, and indicate if changes were made. The images or other third party material in this article are included in the article's Creative Commons license, unless indicated otherwise in a credit line to the material. If material is not included in the article's Creative Commons license and your intended use is not permitted by statutory regulation or exceeds the permitted use, you will need to obtain permission directly from the copyright holder. To view a copy of this license, visit http://creativecommons.org/licenses/by/4.0/
